# Extended perceptive field revealed in humans with binocular fusion disorders

**DOI:** 10.1038/s41598-023-33429-z

**Published:** 2023-04-21

**Authors:** Laura Benhaim-Sitbon, Maria Lev, Uri Polat

**Affiliations:** School of Optometry and Vision Sciences, Faculty of Life Sciences, Bar-Ilan University, Ramat-Gan, Israel

**Keywords:** Visual system, Pattern vision

## Abstract

Binocular vision disorders or dysfunctions have considerable impact on daily visual activities such as reading. Heterophoria (phoria) is a latent eye misalignment (with a prevalence of up to 35%) that appears in conditions that disrupt binocular vision and it may affect the quality of binocular fusion. Our recent study, which used lateral masking (LM), suggests that subjects with binocular fusion disorders (horizontal phoria) exhibit an asymmetry and an abnormal pattern of both binocular and monocular lateral interactions, but only for the horizontal meridian (HM). The perceptive field (PF) is the fundamental processing unit of human vision and both masking and crowding depend on its size. An increased PF size is found in amblyopic populations or in young children. We hypothesized that the PF’s size would be asymmetric only for the phoric group (larger along the HM). We estimated the PF’s size using two different methods (LM with equal-phase and opposite-phase flankers). Phoric subjects exhibited a larger binocular PF size, only for the HM, confirming our hypothesis of an asymmetric PF size. However, the monocular PF size of phoric and control subjects was similar. Phoria affects the PF’s size similarly to meridional amblyopia but without being attributed to abnormal refraction. We suggest that these findings could help explain the inter-observer variability found in the masking literature and the reading difficulties often encountered in subjects with high heterophoria. Since perceptual learning can reduce the PF’s size, further investigation of training may provide a novel therapy to reduce some symptoms related to heterophoria.

## Introduction

Binocular vision refers to a type of vision where the two eyes view a common portion of visual space^1^. Normal binocular vision enables the perception of one single percept (the fusion of the two images from both eyes) and perception in three dimensions (stereopsis)^2,3^; it offers some advantages for performance skills compared with monocular vision, for example, in reaching and grasping^4^, or in reading^5^. Binocular vision disorders or dysfunctions have considerable impact on daily visual activities^6^.

Heterophoria is a common binocular vision disorder frequently encountered in eye care clinics (its prevalence in the adult population is up to 35.6%^7–9^); it is responsible for numerous symptoms and visual discomfort. It consists of a latent misalignment of the eyes, varying with distance, which is revealed in conditions that disrupt binocular vision and may affect the quality of binocular fusion^3^. There are three types of deviation: horizontal, if the visual axis of the eyes converges (esophoria) or diverges (exophoria); vertical, if one visual axis is higher (hyperphoria) or lower (hypophoria) from one another; or cyclorotary, if the eyes are misaligned due to a clockwise or counterclockwise rotation of the eyes (ex- or in cyclophoria). Assessment of heterophoria is critical in clinical practice in order to diagnose non-strabismic binocular dysfunctions, and it is part of the standard tests of binocular vision examinations routinely performed by vision specialists^10,11^. Note that contrary to manifest strabismus, the angle of the phoric misalignment is a reaction to an artificial interference with binocular vision (occlusion of one of the eyes). Although heterophoria is not necessarily pathological, it is often associated with the increasingly prevalent (up to 75% of computers users^12–14^) computer vision syndrome (CVS): a group of eye-related and vision discomfort conditions and symptoms such as headaches, fatigue, or blurry vision, appearing with the prolonged used of display devices^15^. A recent study indicated that patients with heterophoria were more likely to report increased visual discomfort and had less endurance when working on a computer^16^. In reading, it was suggested that the advantage of binocular vision in term of fixation time and reading speed was affected by heterophoria, proportionally to the amount of the heterophoria^5^. Recently, it was suggested that subjects with binocular fusion disorders, such as horizontal heterophoria, exhibit an abnormal pattern of low-level visual processing^17^.

According to the classical understanding of the visual system’s functional structure, the visual information is mediated by neurons via a feedforward network from the retina to the primary visual cortex (V1) where neurons are sensitive to the location, orientation, and spatial frequency of the object presented in their receptive field (RF)^18^. The RF of a simple cell is the specific region of the sensory space (e.g., the visual field) in which a stimulus will modify the firing of that neuron^19^ and it constitutes the fundamental units of visual information analysis. The two monocular RFs are combined to form the binocular RF in V1.

RF processing is not only affected by feedforward stimuli but also by lateral interactions^20–22^ (excitatory or inhibitory signals via the horizontal connections between the ocular dominance column found in V1^23–28^) and by feedback processing^29^. Excitatory or inhibitory interactions between neighboring cortical neurons can either respectively facilitate or suppress the detection of an object and therefore either enhance or diminish one’s perceptual ability^30^. Lateral interactions are revealed by visual masking experiments such as lateral masking (LM). Such experiments show that the perception of a target such as a Gabor Patch (GP) can be either facilitated (enhanced) or suppressed (diminished) when presented between two collinear GP flankers (masks) as a function of the target-to-flankers distance^30,31^. Thus, facilitation appears when the target-to-flankers distance is larger than the RF’s size, whereas suppression is found at shorter distances than the RF’s size^31,32^. In humans, it has been estimated by both theoretical and experimental studies that the optimal shape stimulus for the RF of simple cells to elicit a response is the Gabor patch (Gabor functions, with the standard deviation (*σ*) equal to wavelength (*λ*))^33–35^. The size of the RF is estimated in units of wavelengths (λ), from 2λ to 3λ at the fovea^30,33,36–42^ and it is about 5λ at the periphery at an eccentricity of 4°^32^.

It has been suggested that the RF has a psychophysical correspondent termed the perceptive field (PF) (for a review, see^32,40,43–48^). Whereas the RF is measured physiologically, the perceptive field is measured psychophysically (a perceptual response)^45^. Consequently and analogously to the RF, Lev and Polat^32,40^ suggested that the distance at which the suppression turns to facilitation in the LM experiment is indicative of the PF’s size. Thus, the normal findings regarding the PF’s size correspond to 2–3 λ^30,42,49,50^ in the center of vision and it increases with eccentricity^32,51–53^.

In the literature, few methods have been reported for estimating the PF’s size, leading to similar estimates; among them are studies that used the Westheimer paradigm and the Hermann grid^36,52,54,55^, human and animal physiology^18,47^, and reverse correlation techniques^56^. More recently, Lev and Polat^32,40^ used a lateral masking paradigm suggesting that the PF’s size corresponds to the distance at which suppression turns to facilitation (suppression is maximal when the mask or the surrounding area overlaps with the perceptual field of the target). The estimated size of the PF in this method is in agreement with previous studies using different methods. This method is used to estimate the development of collinear facilitation in children; it is related to the PF’s size^57^; hence, it is the method that we chose for estimating the PF’s size in this study. We compared the estimation of the PF obtained using two types of lateral masking paradigms: the classic LM featuring flankers with an equal phase to the target, and the LM used by Zenger and Sagi^49^ featuring opposite-phase flankers.

The assessment of the PF is of great interest in the theoretical and clinical aspects of vision because it is not only involved in simple object processing in low-level, contrast-based processing^43,44^ such as lines, dots, or bars, with extraction of features such as orientation or spatial frequency^30,58^, but also in more complicated processing of complex scenes^59,60^; thus, it affects the quality of vision. For example, PFs transmit their processing of features to integration fields^61–64^ that are involved in visual crowding^64,65^, a situation whereby it is difficult to discriminate objects or their attributes (like orientation) when surrounded by objects very close to other objects with similar proprieties. In addition, PFs define elements that can be integrated and grouped into a common texture^41,66^.

Thus, PFs are responsible for the processing of potentially large-scale information within fields of flexible size. The PF’s size is modulated by spatial frequency^67^, the intensity of the adapted light level^55,68,69^ and size^64^. It is affected by contrast, target-flanker separation, global configuration, and presentations times^40^. However, the PF’s size can also be modified by perceptual learning^70–72^ or by its development. Recently, it was shown that the binocular perceptive field size decreases with development, reaching an adult’s size in up to 5–6 years^57^. In amblyopia, a pathology resulting from the absence of correlated binocular vision experience during the critical period at about 6 years old, it was shown that the PF’s size was greater than in neurotypical subjects, regardless of the amblyopia type (anisometropic or strabismic)^73,74^. Note that in amblyopia, subjects present a decrease in certain visual abilities such as reduced visual acuity^75–77^, diminished contrast sensitivity^76,78,79^, and especially at high spatial frequencies^61,76,78^, impaired spatial interactions^74^ and a slower reading speed^80,81^.

Benhaim-Sitbon et al.^17^ demonstrated the existence of an abnormal pattern of both monocular and binocular lateral interactions in individuals presenting heterophoria, with an absence of facilitation at 3λ only for the horizontal meridian, pointing out an asymmetry of the lateral interactions. This abnormal pattern resembles the lateral interactions found in strabismic amblyopia^73^. However, the suggestion of a link between the results obtained and the PF’s size in subjects presenting heterophoria called for further investigation. In addition, most previous studies assessing PF size via LM experiments generally used vertical lateral masking, which provides information about PF size corresponding to the vertical meridian^32,40,57^. To date, a comparison of the PF along the horizontal and the vertical meridians has not been investigated, in general, and in heterophoria, in particular. Considering the above, we hypothesized that the PF’s size would be larger along the horizontal meridian only for the heterophoric group due to the latent eye misalignment and its impact on binocular fusion. This research aims to assess the PF’s size at different viewing distances, the symmetry of the PF (horizontal versus vertical), and monocular versus binocular PF size, and to compare these results using two different methods (LM with equal-phase and opposite-phase flankers).

Importantly, we found a larger binocular PF size only for the horizontal meridian of the heterophoric group. This indicates an asymmetry of the PF for subjects presenting heterophoria: it is larger on the horizontal meridian than on the vertical meridian. This larger PF size along the horizontal meridian resembles the increased PF size found in amblyopia without being attributed to a refractive error or manifest strabismus. The horizontal monocular PF was smaller than the horizontal binocular PF within the heterophoric group. Although it was previously shown that the monocular lateral interactions differed between the controls and the heterophoric group for the horizontal meridian^17^, we did not find any difference between the monocular PF sizes of the two groups.

## Methodology

### Subjects

Twenty-five subjects participated in 3 experiments in the study (some participated in more than one experiment, see Table 1 for details), aged from 19 to 35 years old (27.36 ± 4.94, mean ± STD). Each subject was included after a comprehensive orthoptic examination by a certified orthoptist including both sensory and motor assessments. The procedures of the orthoptic assessment were the same as in a previous study and are fully detailed in the Supplementary Material: 10.1038/s41598-022-16458-y, except for the subjects in experiment 3 whose stereoacuity was measured by the Randot Stereotest (Stereo Optical, Inc., Chicago, IL) and the angle of phoria measured by the alternating cover test (ACT) only. All observers had normal or corrected-to-normal visual acuity (both monocular and binocular), normal stereoscopic vision (a minimum of 40 arcsec) and fusion at all viewing distances. Subjects with horizontal phoria equal or superior to 6Δ (prism diopters), and/or vertical phoria equal or superior to 2Δ at least at one testing distance (distance or near) were attributed to the heterophoric group if they did not reveal any decompensation to intermittent strabismus. All subjects did not show any clinical signs of accommodative disorders, amblyopia, stereopsis disorders, strabismic problems, any decompensation to intermittent strabismus, or ocular disease (exclusion criteria for both groups).

**Table 1:** Clinical orthoptic details on the enrolled subjects.

Subject	Gender	Age	Group	Cover test				Maddox				Dominant eye	Stereoacuity (arcsec)	Step convergence (break) in Δ		Step divergence (break) in Δ		NPC (cm)	Experiments
				4 m	1 m	60 cm	40 cm	4 m	1 m	60 cm	40 cm			4 m	40 cm	4 m	40 cm		
S1	F	34	Phoria	E10	E8	E6	E′6	E10	E8	E6	E′6	OD	20	40	40	2	4	3	All
S2	F	27	Phoria	ortho	ortho	E2	E′6	ortho	E1	E2	E′6	OD	40	18	40	2	4	4	1, 2
S3	F	19	Phoria	X4	X4	X10	X′16	X4	X4	X8	X′12	OD	40	14	8	6	14	9	1, 2
S4	M	33	Phoria	X2	X3	X7	X′10	X2	X3	X7	X′10	OD	30	18	10	4	12	12	All
S5	M	33	Phoria	X2	X4	X6	X′8	X2	X4	X6	X′8	OS	20	20	18	4	12	10	All
S6	M	24	Phoria	X2	X2	X2	X′14 RH′2	X2	X2	X2	X′14 RH′2	OD	40	20	18	6	16	10	1, 2
S7	M	34	Phoria	ortho	ortho	X2	X′6	X2	X2	X4	X′8	OD	40	8	8	4	12	13	1, 2
S8	F	32	Phoria	ortho	ortho	X2	X′6	E2	E2	E2	E′2	OD	40	20	20	4	8	10	1, 2
S9	F	22	Control	X4	X4	-	X′4	X4	X4	-	X′4	OS	40	18	35	4	12	5	1
S10	F	28	Control	ortho	X2	X2	X′4	ortho	X2	X2	X′4	OD	40	20	40	6	14	5	1, 2
S11	M	26	Control	ortho	ortho	ortho	ortho	E1	E1	ortho	ortho	OD	40	25	35	6	16	4	1, 2
S12	F	33	Control	ortho	ortho	ortho	X′2	ortho	ortho	LH1	LH′1	OD	40	14	25	4	12	5	1, 2
S13	F	23	Control	ortho	ortho	X2	X′5	ortho	ortho	X2	X′5	OD	40	18	30	4	8	4	1, 2
S14	F	20	Control	X2	X2	X2	X′5	X2	X2	X2	X′5	OD	40	18	30	4	10	5	2
S15	F	35	Control	ortho	ortho	ortho	X′2	ortho	RH1	RH1	RH′1	OD	40	14	25	6	12	5	1, 2
S16	M	33	Control	ortho	ortho	ortho	ortho	ortho	X2	X2	X′4	OS	40	14	20	4	12	7	1, 2
S17	M	23	Control	ortho	ortho	ortho	ortho	ortho	ortho	ortho	X′1	OS	20	12	20	4	10	10	1,2, 3A
S18	F	28	Phoria	X10	X12	–	X18	–	–	–	–	OD	30	8	10	4	18	20	3A and 3B
S19	F	23	Phoria	X6	X6	–	X14	–	–	–	–	OS	30	10	14	4	16	5	3A and 3B
S20	F	31	Control	ortho	ortho	–	X2	–	–	–	–	OD	20	30	40	4	12	3	3A and 3B
S21	F	24	Control	ortho	ortho	–	E2	–	–	–	–	OD	20	10	20	6	10	5	3A
S22	F	25	Control	ortho	ortho	–	ortho	–	–	–	–	OD	20	25	30	4	8	3	3A
S23	F	23	Control	X2	X2	–	X5	–	–	–	–	OD	20	12	18	4	8	10	3A and 3B
S24	F	23	Control	X2	X2	–	X4	–	–	–	–	OD	20	6	18	4	10	5	3B
S25	M	28	Control	ortho	ortho	–	ortho	–	–	–	–	OS	30	10	14	4	12	5	3B

*F* female, *M* male, *ortho* orthophoria, *X* exophoria, *E* esophoria, *RH* right hyperphoria, *LH* left hyperphoria, *OD* right eye, *OS* left eye, *NPC* near point of convergence, *Δ* prismatic diopter, *cm* centimeters.

The study protocol was approved by the Internal Review Board (IRB) of Bar-Ilan University. Informed consent was obtained from all subjects and/or their legal guardian(s). All methods were performed in accordance with the relevant guidelines and regulations.

### Apparatus and stimuli

For stimuli presentation, we utilized a PC controlled by a NVDIA GTX 710 video card and a BENQ XL 2411 color monitor using an in-house-developed software for psychophysical experiments (PSY) developed by Bonne^82^. The screen resolution was 1920 × 1080 pixels, and gamma correction was applied.

**Figure 1:**
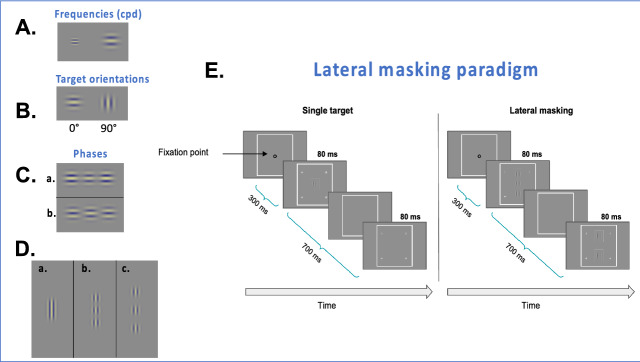
(**A**) Illustration of the two different frequencies used in Experiment 3: on the left, a Gabor Patch (GP) of 8 cpd (cycles per degree), and on the right, a GP of 4 cpd. (**B**) Illustration of the single-target orientations. (**C**) Illustration of a central GP target flanked by a pair of GP masks with (a) an equal phase pattern and (b) an opposite phase pattern. (**D**) Examples of some target-to-flanker separations. (a) 1.5λ (b) 3λ, and (c) 6λ. (**E**). The lateral masking paradigm used for the sets of experiments. Observers had to report in which interval (the first or the second) the central Gabor target appeared. The contrast in all the targets was enhanced for demonstration purposes.

Stimuli were localized gray-level gratings (Gabor patches) with equal wavelength (λ) and standard deviation (STD, σ), allowing a minimum of 2 cycles in the Gabor patches. We used two spatial frequencies (see Fig. 1A): 4 and 8 cycles per degree (cpd, λ = 0.433° and 0.21°, respectively). The Gabor patches were modulated from a background luminance of 40 cd/m^2^.

### Procedures

Three different sets of experiments were performed (for a summary of the experimental protocol, see Table 2). For all the experiments, we used a standard contrast detection task under lateral masking (LM) conditions. The Gabor Patches were presented following two global orientations (0 and 90 degrees, see Fig. 1B). For each orientation, two conditions were tested (Fig. 1E): a single Gabor Patch (sGP) contrast detection threshold and a sGP contrast detection threshold in the presence of two collinear GP flankers having a contrast of 60% (LM). Different target-to-flankers separations (λ) were employed (see Fig. 1D). To measure the central GP target contrast threshold, we used a two-alternative temporal forced-choice paradigm and a 3:1 staircase procedure (converging to 79% correct responses)^83^.

**Table 2:** Experimental protocols.

Experiments	Tasks	Target- flankers separations (λ)	Flankers phases	Viewing distance (m)	Viewing modes	Orientations	Nmax trials	N repetitions	SF (cpd)	Method	Presentation time (ms)	Total number of subjects (n)
1	CD + LM	2,3,4,6	Equal	0.4, 0.6, 1	Binocular	90°, 180°	80	2	4	2AFC	80	16
2	CD + LM	2,3,4,6	Equal	1	Binocular + monocular	90°, 180°	80	2	4	2AFC	80	16
3A	CD + LM	1.5, 2, 2.5, 3, 3.5, 4, 4.5, 5	Equal + opposite	1	Binocular	90°, 180°	80	3	4	2AFC	80	10
3B	CD + LM	1.5, 2, 2.5, 3, 3.5, 4, 4.5, 5	Equal + opposite	1	Binocular	90°, 180°	80	3	8	2AFC	80	8

*CD* contrast detection, *LM* lateral masking, *N* number, *AFC* alternating forced choice.

The subjects were instructed to maintain their fixation at the center of a screen denoted by a small circle at the beginning of each trial and to avoid eye movements during the trials (blinking was permitted). When ready, they pressed the middle button of the mouse to activate a trial sequence: a no-stimulus interval 300 ms with a temporal jitter of 500 ms (0–500 ms with equal distribution) and two sequential stimulus presentations (80 ms each) that were separated by another no-stimulus interval (700 ms + temporal jitter up to 500 ms. Only one of the two randomly ordered stimulus presentations contained the target; for the LM condition, both contained the mask. The GP presentations (stimulus intervals) were indicated by four peripheral high-contrast crosses. Subjects had to report which of the two stimulus presentations contained the target GP by pressing a mouse button (left for the first one and right for the second one). Auditory feedback was given for incorrect responses.

The GP amplitude and the distance between the target and flankers were kept constant for each trial. Screen luminance remained the same during the stimulus and the no-stimulus intervals. Throughout all the experiments, a peripheral lock was used to limit phoria decompensation during the absence of stimuli (and/or the interstimulus interval). The peripheral lock consisted of a square sustaining 15.4° visual degrees horizontally and vertically from the center.

Experiments were run during either binocular viewing (the two eyes seeing simultaneously) or monocular viewing (one eye was occluded by a transparent diffuser). For a summary of the entire experimental design, see Table 2.

#### Experiment 1: Binocular perceptive field assessment for horizontal and vertical meridians at different sitting distances

It has been suggested^30,49^ and found in a previous study^40^ that the suppressive zone obtained in the LM experiment corresponds to the size of the perceptive field. In our recent study^17^, we found that the suppressive zone was larger only for the horizontal meridian in subjects presenting a high heterophoria. We decided to investigate whether the horizontal binocular PF is greater than the vertical one for this population. In addition, since the angle of the phoria can change at near or far distances, we wanted to evaluate the PF’s size for different working distances. The subjects sat at three different viewing distances: 40 cm (the usual reading distance), 60 cm (the usual working distance from a computer) and at one meter. The experiment consisted of contrast detection of a single target (Gabor patch), followed by lateral masking with target-to-flanker separations (λ) of 2λ, 3λ, 4λ, and 6λ. Thus, for each GP orientation (0° and 90°), the experiment consisted of 5 blocks (sGP contrast detection and LM by 2λ, 3λ, 4λ, and 6λ). Each condition was repeated twice; thus, each subject ran a total of four tests of LM for each viewing distance. Depending on the attentional capacity and fatigability of the observer, up to eight sessions were needed to collect all the data when each session lasted from 1 to 2 h to run a total of 60 blocks.

The peripheral lock, GP frequencies, and cross sizes were modified according to the viewing distance so that the experimental parameters were identical; the viewing distance and the angle of the heterophoria were the only parameters that varied.

#### Experiment 2: Monocular and binocular perceptive field assessment

Since heterophoria is a binocular fusion disorder, we wanted to compare the monocular and the binocular perceptive fields. The observers sat at 1 m from the computer screen, and each subject’s dominant and non-dominant eye were tested separately. To avoid binocular rivalry, we occluded the non-tested eye with a frosted diffuser. For a complete summary of the experimental design, see Table 2.

#### Experiment 3: Assessment of the binocular perceptive field with equal and opposite phase flankers

In order to verify the PF assessment, we used another method of assessment consisting of masking the central GP target by a pair of Gabor signals with opposite phase patterns and comparing it with results in which the GP was masked by flankers with equal phases (see Fig. 1C). First, this set of experiments was conducted with a GP frequency of 4 cpd (designated as Experiment 3A). The experimental parameters were identical to those of Experiment 1 except for the repetition and the target-to-flanker separations. Each experiment was repeated three times. We used 8 different target-to-flanker separations (1.5 λ, 2 λ, 2.5 λ, 3 λ, 3.5 λ, 4 λ, 4.5 λ, and 5 λ) so that each subject ran 27 blocks for each orientation. Up to five meetings of one to two hours were needed to collect the data for a total of 54 blocks per subject.

Since the contrast detection thresholds of a single GP of 4 cpd were low, we decided to run an additional sub-experiment (Experiment 3B) with a GP frequency of 8 cpd to prevent any potential floor effect of the facilitation (see Fig. 1A). All other parameters were identical to those in experiment 3A. For details of the experimental protocol, see Table 2.

### Estimation of the perceptive field size

Lev and Polat^32,40^ suggested that the distance at which the suppression turns to facilitation provides an estimate of the size of the PF. For each subject, we used the crossover point where collinear suppression was transformed to facilitation (y = 0) as the crossing border of the PF^32,40,57^. When the data could not be extracted from the results, we extrapolated the results assuming a suppression of 0.15 log units at 1 λ according to the findings in the literature^30,40^.

### Data and statistical analysis

Repetitions for each condition were averaged for each subject.

A Welch two sample t-test with equal variance was performed to test the effect on one nominal variable (group) for a continuous outcome (the perceptive field size).

Pearson’s and Spearman’s correlation tests were conducted between the perceptive field size and the angle of the phoria.

We used a two or a three-way mixed ANOVA to test the effect of 2 or 3 nominal variables (such as group, viewing distance, viewing mode, mask phase, and meridian) on continuous outcomes (the perceptive field size). Linear mixed effect models were performed, and the ANOVA was completed on the resulting models. We defined all nominal variables as fixed effects, and subject ID as a random effect. All interactions were included in the initial models however, if the interactions were non-significant, we refitted the models without these interactions. We performed a post-hoc analysis as pairwise comparisons defined by linear contrasts. FDR correction was applied to control for multiple testing. If the interactions were removed, we performed the post-hoc analysis by averaging the non-interacting factors. We checked graphically the normality of the residual homogeneity of variance assumptions.

All data points were confirmed for not being outliers.

## Results

### Experiment 1: Binocular perceptive field assessment for horizontal and vertical meridians at different sitting distances

#### Subjects

Sixteen subjects participated (8 in the heterophoric group and 8 in the control group) in the experiment. The average age in the control group was 27.6 ± 4.6 years old (mean ± STD), and in the heterophoric group it was 29.5 ± 5.5 years old (mean ± STD). All subjects had a stereoacuity of 40 arcsec. No subject reported a double vision or intermittent double vision during the experiment. Detailed results such as heterophoria measurements for the subjects can be found at 10.1038/s41598-022-16458-y.

For 4 subjects at 40 cm (2 from the control group and 2 from the heterophoric group) and for 2 subjects at 60 cm (one phoric subject and one control), the crossover point where the facilitation zone is transformed into the suppression zone could not be extracted from the data at 2λ (there was facilitation, i.e. a negative elevation threshold). Thus, we extrapolated the data following the method described above (see “Estimation of the perceptive field size”).

#### Contrast detection threshold for single targets

The contrast detection thresholds for single targets were not statistically different between the two groups for each distance. The values of the contrast detection thresholds obtained are presented in Table 3. We performed a three-way ANOVA to determine the effect of group, target orientation, and viewing distance on the contrast detection threshold. The average contrast detection threshold was statistically similar between the control and the heterophoric groups (F (1,14) = 0.4345, *p* = 0.5205) nor between the two target orientations (F (1,70) = 0.0035, *p* = 0.9531). The viewing distance had a constant effect on the contrast threshold (F (2,70) = 50.2708, *p* < 0.0001); however, no significant interaction was revealed. We note that the contrast detection thresholds obtained at 40 cm for both orientations and groups were statistically higher than at 1 m as reported in a previous study^17^ (post-hoc analysis).

**Table 3:** Values of contrast detection threshold (in log unit) obtained for each group for the different target orientations at different viewing distances (Experiment 1). H stands for horizontal and V for vertical.

Groups	Target orientations	Contrast thresholds (log unit)		
		40 cm	60 cm	1 m
Controls	H	0.781 ± 0.012	0.628 ± 0.052	0.577 ± 0.031
	V	0.791 ± 0.016	0.614 ± 0.054	0.548 ± 0.037
Heterophoria	H	0.810 ± 0.024	0.651 ± 0.048	0.565 ± 0.031
	V	0.791 ± 0.032	0.672 ± 0.050	0.590 ± 0.047

#### Horizontal and vertical binocular perceptive field size

**Figure 2:**
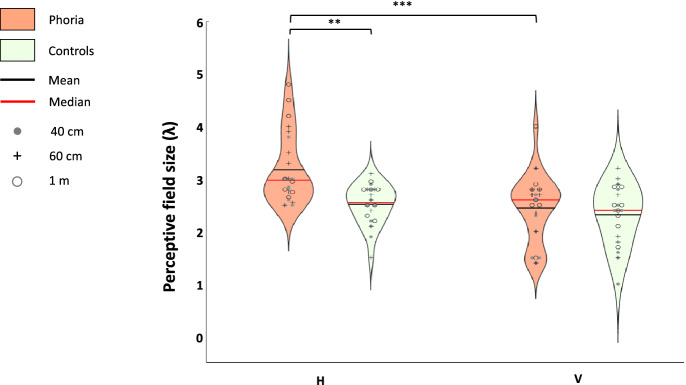
The violin plots represent the distribution of the perceptive field size for the horizontal (H) meridian (the orientation of a target of 0°) and the vertical (V) meridian (the orientation of a target of 90°). The phoria group (n = 8) is denoted in dark orange and the control group (n = 8) is denoted in light green. The results were obtained in Experiment 1. Each violin plot contains the results of all three viewing distances. ***p ≤ 0.001, **p ≤ 0.01, *p ≤ 0.05.

We assessed the PF’s size for the horizontal and vertical meridians at 40 cm, 60 cm, and 1 m viewing distances for both groups. We performed a three-way ANOVA to test the effect of group, viewing distance, and meridian on the PF’s size. Group (F (1,25.7163) = 8.4093, *p* = 0.0075) and meridian (F (1,76.2332) = 20.68.33, *p* < 0.0001) had a constant effect on the PF’s size. The viewing distance (F (2,76.2332) = 5.0565, *p* = 0.0087) had a significant effect on the PF’s size, which does not depend on the group or meridian. However, we found a significant interaction between group and meridian F (1, 76.2332) = 6.9383, *p* = 0.0102). Thus, the post hoc analysis includes comparisons between all sitting distances (disregarding other factors—i.e., averaging the group and meridian), and comparisons between group and meridian (averaging the sitting distances). The results are summarized in Fig. 2. The post-hoc analysis revealed that, when averaging the viewing distances, the horizontal PF’s size was (3.175λ ± 0.65, mean ± SE), which is statistically larger than the vertical PF’s size (2.44λ ± 0.65, mean ± SE), only within the phoria group (*p* < 0.0001) but not within the control group (horizontal PF: 2.51λ ± 0.51, vertical PF: 2.32λ ± 0.47, mean ± SE, p = 0.2519). In addition, the horizontal PF’s size was statistically larger for the heterophoric group than for the control group (a mean difference of 0.735λ, p = 0.0016). The size of the vertical PF was similar between the two groups (an insignificant mean difference of 0.19λ, p = 0.3975). When comparing the PF’s size obtained for each sitting distance disregarding the other factors (i.e., averaging the group and meridian), we found that the PF’s size at 40 cm was statistically smaller than at one meter distance (a mean difference of 0.455, p = 0.0158). We suggest that this occurs because the contrast detection thresholds of the single target were higher at 40 cm than at one meter as reported in a previous article^17^.

#### The relationship between the binocular PF for the horizontal meridian and the amount of phoria

We tested the relationship between the binocular PF for the horizontal meridian and the amount of phoria in absolute values (both the horizontal directions of misalignment) for all subjects. We included all subjects that underwent a lateral masking with equal phase with a GP of 4 cpd, in total, 23 subjects (subjects of experiments 1, 2, and 3A). There was a low correlation between the size of the PF obtained at one meter viewing distance and the amount of phoria with the Spearman’s correlation test (rho = 0.27); however, it was significant (p = 0.049).

### Experiment 2: Monocular and binocular perceptive field assessments

Experiment 1 showed a clear asymmetry of the binocular perceptive field between the horizontal and the vertical meridians as well as a binocular horizontal PF of a greater size for the heterophoric group. Since heterophoria is a binocular sensorimotor disorder and since it previously suggested the existence of an abnormal pattern for the monocular lateral interactions along the horizontal meridian in heterophoria (very poor facilitation at 3λ, significantly different from the control group)^17^, we wanted to compare the binocular and the monocular perceptive fields for the horizontal meridian within the heterophoric group.

#### Subjects

The data were collected from sixteen subjects. The eight subjects from the heterophoric group were the same as in experiment 1; seven subjects in the control group participated in experiment 1, and one new control subject was added (see Table 1). All subjects had a stereoacuity of 40 arcsec. Details about the subjects can be found in “Results” 10.1038/s41598-022-16458-y. No participant reported a double vision or intermittent double vision during the experiment.

#### Contrast detection threshold for single targets

The contrast detection thresholds for single targets were not statistically different between the two groups for each target orientation and eye-viewing trial. The values of the contrast detection thresholds obtained are presented in Table 4. A three-way ANOVA was performed to test the effect of target orientation (horizontal or vertical), group, and eye viewing (dominant, non-dominant, or binocular viewing) on the contrast detection threshold. We noted a a constant effect of the type of eye viewing on the contrast threshold (F (2,70) = 17.2195, *p* < 0.0001). A post-hoc analysis revealed that, within the control group, the average contrast threshold obtained for the non-dominant eye (0.87 ± 0.07 log units, mean ± SE) was significantly higher than during binocular viewing (0.57 ± 0.03 log units, mean ± SE) only for the vertical condition (*p* = 0.0007), which was in agreement with earlier studies^30,31^. Within the heterophoric group, no significant difference of contrast threshold was noted for both the vertical and the horizontal conditions.

**Table 4:** Values of contrast detection threshold (in log units) obtained for each group for the different target orientations during different types of eye viewing (Experiment 2). H stands for horizontal and V for vertical orientation.

Groups	Target orientations	Contrast thresholds (log unit)		
		Dominant eye	Non-dominant eye	Binocular
Controls	H	0.775 ± 0.055	0.77 ± 0.05	0.61 ± 0.05
	V	0.74 ± 0.06	0.82 ± 0.07	0.56 ± 0.03
Heterophoria	H	0.71 ± 0.05	0.685 ± 0.03	0.56 ± 0.03
	V	0.62 ± 0.06	0.65 ± 0.04	0.59 ± 0.05

#### Monocular and binocular perceptive field size

First, we wanted to compare the PF’s size from both eyes. We performed a three-way ANOVA to test the effect of group (F (1,22.5714) = 3.5767, *p* = 0.0715), meridians (F (1,42.50006) = 0.0011, *p* = 0.9734), or eye (F (1,42.50006) = 0.0451, *p* = 0.8328) on the monocular PF’s size. Since no significant effect or significant interactions (p-values were between 0.2135 and 0.9203) were revealed, we averaged both monocular PF sizes for each subject.

Within the phoria group, the horizontal binocular PF values (3.41λ ± 0.31, mean ± SE) were larger than those of the monocular PFs (2.46λ ± 0.22, mean ± SE, t(2.6), *p* = 0.0213), whereas for the control group, there was no statistical difference (horizontal binocular PF: 2.45λ ± 0.15, horizontal monocular PF: 2.08λ ± 0.12, mean ± SE, t(1.8), *p* = 0.0898). Nevertheless, the horizontal monocular PF size between the controls and the phoria groups was statistically similar (a mean difference of 0.0187λ, t(− 1.5), *p* = 0.1755). Analogously to experiment 1, the horizontal binocular PF was statistically larger for the heterophoric group than for the controls (a mean difference of 1.0062λ, t(2.8), *p* = 0.0245).

### Experiment 3: Assessment of the binocular perceptive field with equal and opposite phase flankers

In the previous experiments, we used GP masked by flankers with equal phase and determined the border of PF as the crossover point where collinear suppression is transformed to facilitation (y = 0). For this experiment we decided (a) to add more target- flanker distances to determine more precisely the crossover point and therefore the size of the PF, and (b) to compare the results obtained with equal-phase flankers to another method (flankers with an opposite phase). Until this point, the previous experiments indicated that the horizontal binocular PF of the phoric group is larger than (a) the horizontal binocular PF for the control group, (b) the vertical binocular PF of the phoric group, which showed an asymmetry of the PF, and (c) the horizontal monocular PF of the phoric group. However, the controls and the phoric group had a statistically similar size of the horizontal monocular PF.

We wanted to better define the limit of the PF’s size using a lateral masking experiment with flankers having an opposite phase to each other. This experiment was conducted in two phases: the first one contained GPs of 4 cpd (experiment 3A) and the second one contained GPs of 8 cpd (termed experiment 3B). Since the contrast thresholds of the single target obtained with a GP of 4 cpd were low, it may limit the amount of facilitation observed (the floor effect). Thus, we decided to repeat the experiment with a GP of 8 cpd; it had a higher contrast detection threshold and a smaller effective size of the GP (because our GP has an equal wavelength (λ) and a standard deviation (σ), an 8 cpd GP appears smaller than a 4 cpd GP). Hence, the facilitation limits should be sharper and the potential difference in the PF size should be better highlighted. This experiment was run under binocular viewing; therefore, the “PF” mentioned corresponds to the binocular PF.

#### Subjects

Twelve subjects participated in this experiment. For sub-experiment 3A, five controls and five subjects with high heterophoria participated, and for sub-experiment 3B, there were eight subjects (four controls and four phoria). Two controls differed in the two sub-experiments. The average age in the heterophoric group was 30.2 ± 4.6 years old (mean ± STD), and in the control group it was 25.6 ± 3.7 years old (mean ± STD) in experiment 3A, and 25.2 ± 3.3 years old (mean ± STD) in experiment 3B. The angle of deviation of the phoric group was larger than for the control group at 40 cm (group: mean ± SE, heterophoria: 11.2Δ ± 1.92, controls: 1.80Δ ± 0.81, p = 0.0135, unpaired two-tailed t-test) and at 1 m (group: mean ± SE, heterophoria: 6.60Δ ± 1.43, controls: 0.4Δ ± 0.35, p = 0.0212, unpaired two-tailed t-test). Within the phoria group, one participant presented esophoria and the four other subjects presented exophoria. Each participant had a normal stereoacuity between 20 and 30 arcsec, and no participant reported double vision or intermittent double vision during the experiment.

#### Contrast detection threshold for single targets

Since the different phases could not impact the contrast of the single target (flankers appear only during the lateral masking experiment), we averaged for each subject and each target orientation the contrast threshold obtained for the single target for an equal-phase LM and an opposite phase LM. A paired t-test confirmed that there was not any statistical difference (HM: p = 0.7091, VM: p = 0.9127). Therefore, we tested the effect of group and target orientation on the contrast detection threshold by using a two-way ANOVA.

##### Experiment 3A

The contrast thresholds were statistically similar between the two groups (F (1,8) = 3.5026, *p* = 0.09818). The target orientations (F (1,8) = 3.5049, *p* = 0.09808) did not have any significant effect on the contrast threshold, nor did the interaction between group and target orientation (F (1,8) = 4.8778, *p* = 0.05821). The average values of contrast threshold detection obtained are summarized in Table 5.

**Table 5:** Values of contrast detection threshold (in log units) obtained for each group for the different target orientations during different types of eye viewing (Experiment 3).

Group	Target orientations	Contrast thresholds (log unit)	
		Exp. 3A	Exp. 3B
Controls	H	0.56 log unit ± 0.06	0.90 ± 0.08
	V	0.46 log unit ± 0.02	0.995 ± 0.08
Heterophoria	H	0.42 log unit ± 0.02	0.80 ± 0.06
	V	0.40 log unit ± 0.01	0.83 ± 0.02

*H* stands for horizontal, *V* for vertical orientation, and *Exp.* for experiment.

##### Experiment 3B

The contrasts were statistically similar between the two groups (F (1,6.052) = 3.8959, *p* = 0.09543). The target orientations (F (1,12.049) = 0.0292, *p* = 0.86710) did not have any significant effect on the contrast threshold. No significant interaction was observed between group and target orientation (F (1, 12.049) = 1.4518, *p* = 0.25137). The average values of contrast threshold detection obtained are summarized in Table 5.

#### Binocular perceptive field size with equal and opposite phase flankers

##### Experiment 3A

**Figure 3:**
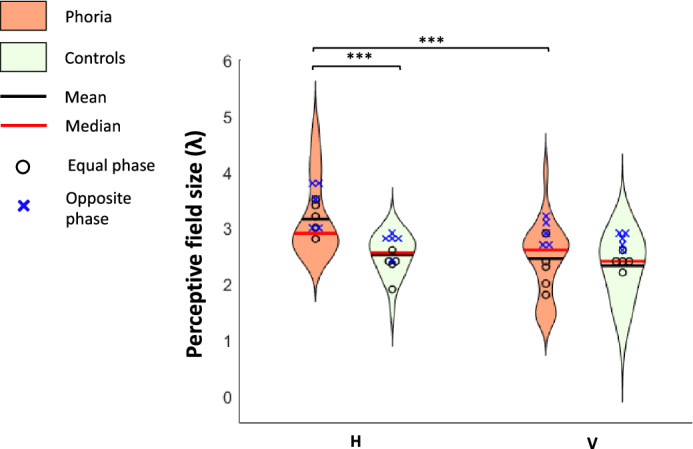
The violin plots represent the distribution of PFs for the horizontal meridian (H) and the vertical meridian (V). Data for the phoric group (n = 5) are denoted in dark orange and for the controls (n = 5) data are denoted in light green. The PF sizes obtained when the target was masked by equal-phase flankers are denoted by a black circle, and the PF sizes obtained when the target was masked by opposite-phase flankers are denoted by a blue cross. ***p ≤ 0.001, **p ≤ 0.01, *p ≤ 0.05.

We measured the PF’s size obtained with both equal-phase and opposite-phase flankers for both groups and meridians (vertical and horizontal). The results are presented in Fig. 3. We used a three-way ANOVA to evaluate the effect of group, meridian, and phase pattern on the PF’s size. Group (F (1,35) = 19.712, *p* < 0.0001) and meridian (F (1,35) = 13.653, *p* = 0.0007) had a constant effect on the PF’s size. Phase (F (1,35) = 22.882, *p* =  < 0.0001) had a significant effect on the PF’s size, which does not depend on the group or meridian. However, we found a significant interaction between the group and meridian (F (1, 35) = 18.707, *p* = 0.0001). Since the phase did not have any significant interaction, we averaged the PF’s size obtained by both methods. A post-hoc analysis revealed that within the phoric group, the horizontal PF’s size (3.3λ ± 0.11, mean ± SE) was significantly larger than its vertical PF (2.6λ ± 0.14; mean ± SE*, p* =  < 0.0001) and the horizontal PF of the control group (2.535λ ± 0.09, mean ± SE, *p* =  < 0.0001).

##### Experiment 3B

We measured the PF’s size obtained through a lateral masking experiment presenting GPs of 8 cpd with equal-phase and opposite-phase flankers for both groups and meridians (vertical and horizontal). We performed a three-way ANOVA to test the effect of group, meridian, and phase on the PF’s size. Phase (F (1,18) = 18.4388, *p* < 0.0001) and meridian (F (1,18) = 7.8183, *p* = 0.0119) had a constant effect on the PF’s size. Group (F(1,6) = 3.9, *p* = 0.0940) did not have a significant effect on the PF’s size. However, we found a significant interaction between group and meridian (F (1, 18) = 6.2327, *p* = 0.0224) and between group and phase (F (1, 18) = 5.7440, *p* = 0.0276). Using a post-hoc analysis, we found that the horizontal PF was statistically different in the control and the phoric group only with an opposite phase (group: mean ± SE, phoria:3.45λ ± 0.18, controls: 2.40λ ± 0.20, p = 0.0067). Within the phoric group only, the PF’s size was larger with opposite-phase than with equal-phase flankers for the horizontal meridian (phase: mean ± SE, equal:2.775λ ± 0.25, opposite: 3.45λ ± 0.18, *p* = 0.0443) and for the vertical meridian as well (phase: mean ± SE, equal: 1.95λ ± 0.15, opposite: 2.95λ ± 0.20, *p* = 0.0067). For the control group, the PF’s size was not larger with an opposite phase than with an equal phase for the horizontal meridian phase: (mean ± SE, equal: 2.50λ ± 0.25, opposite: 2.40λ ± 0.20, *p* = 0.6943). For the vertical meridian, the PF’s size was larger with opposite-phase flankers but not significantly (phase: mean ± SE, equal: 2.125λ ± 0.16, opposite: 2.7λ ± 0.12, *p* = 0.0812). We found an asymmetry of the PF only for the phoria group, when assessing the PF by equal-phase flankers (a mean difference of 0.825λ, *p* = 0.0161) but not with opposite-phase flankers (a mean difference of 0.5λ,* p* = 0.1223).

Within the phoric group and for the horizontal meridian, we noted a bigger difference between the horizontal PF’s size obtained with GPs of 8 cpd than with 4 cpd for equal-phase flankers (frequency: mean ± SE, 8 cpd: 2.7λ ± 0.25, 4 cpd: 3.18λ ± 0.14, *p* = 0.2184) than for opposite-phase flankers (frequency: mean ± SE, 8 cpd: 3.45λ ± 0.1848, 4 cpd: 3.42λ ± 0.20), although this difference was not statistically significant (unpaired t-test with unequal variance, respectively,* p* = 0.2184 and *p* = 0.9107).

## Discussion

Binocular vision enables a vision of a single percept by the mechanism of fusion and in three dimensions by mechanisms related to retinal disparity. Binocular vision dysfunction will have a negative impact on daily visual activities such as reading or working on the computer (with CVS). Heterophoria is a common type of binocular disorder; it consists of latent misalignment of the eyes with potential consequences on the quality of the binocular fusion.

Since we recently have shown that there was an abnormal pattern and an asymmetry of the lateral interactions in subjects presenting high heterophoria^17^, we hypothesized that the PF’s size may be asymmetric in this population, with a larger PF for the horizontal than for the vertical meridian. We investigated how heterophoria would impact the PF’s size during both binocular and monocular viewings, at different viewing distances and for different stimulus frequencies. We found that the binocular PF’s size was larger for the heterophoric group than for the control group, but only for the horizontal meridian. These results are in agreement with the expectation for asymmetry of the PF’s size for subjects presenting heterophoria, and it resembles the PF size found in populations with visual developmental disorders such as amblyopia, but here they were not attributed to abnormal refraction or manifest strabismus. These findings were consistent between two methods of estimating the PF (LM with equal-phase and opposite-phase flankers) and when varying the frequency of the stimulus.

### Horizontal and vertical binocular PF: an asymmetry

Recently, it was shown that the binocular perceptive field size decreases with development, reaching an adult’s size in up to 5–6 years^57^; however, to date, no study has investigated the symmetry of the PF along the horizontal and vertical meridian, since lateral masking experiments are mainly run with vertical configurations^30–32,40^. An estimation of the PF’s size revealed that subjects with a high horizontal heterophoria exhibit a binocular horizontal PF size that is 30.1% larger than their binocular vertical PF. When the frequency of the target was increased, we even found that the horizontal binocular PF’s size was up to 42.5% larger than the vertical PF’s size. This is in agreement with the hypothesis of the asymmetry of the PF for heterophoric people. Interestingly, for control subjects, we also observed an asymmetry of the PF; however, it was reduced, compared with the phoric group (the horizontal PF’s size was 8% larger than that of the vertical meridian and 17% larger when the frequency of the target was increased). Note that for control subjects only, with a frequency of 8 cpd and with opposite-phase flankers only, we found a decrease in the PF’s size for the horizontal meridian of about 12%. Since both masking and crowding are dependent on the perceptual field size^40,57,73,84^ and crowding and reading are linked via the critical spacing^85^, we suggest that this increase in the PF’s size only for the horizontal meridian could be responsible for the difficulties found in reading^5^, for some symptoms in CVS^86^, or for some difficulties in adjusting virtual reality devices. In addition, we suggest that this asymmetry of the PF’s size corroborates the asymmetry of crowding reported in some previous studies^40,65^.

### Abnormal binocular horizontal PF in heterophoria resembles pseudo-meridional amblyopic behavior

In the heterophoric group, the PF’s size in binocular viewing was about 26.5% larger than for the controls, but only for the horizontal meridian, thus confirming our hypothesis. The vertical binocular PF’s size was statistically similar between the two groups (we noted an insignificant increase of 5% for the heterophoric group). This result may explain the suppression that we found at 3λ in a previous study^17^. Larger PF sizes are also found in amblyopia^73,74^, a default in maturation of the visual system due to refractive disorders or strabismus. Amblyopia is characterized by a number of behavioral, neural, perceptual, oculomotor, and clinical abnormalities (for a review, see^84,87^). This larger horizontal PF size found in the heterophoric group resembles what a PF would be in meridional amblyopia, without being attributed to abnormal refraction. Recently it has been shown that the PF’s size varies with the development: about 5λ at age 3, 4λ at 5 years old, to the normative value of 3λ at about 5.5–6 years old. Thus, these findings suggest a meridional developmental disorder in case of high heterophoria. If so, it would be interesting to further investigate perceptual learning for the horizontal meridian only. Indeed, it has been suggested that perceptual learning reduces the size of the human perceptive field^88^, and it has been shown that perceptual learning using a lateral masking paradigm enables remarkable restauration of certain visual abilities in amblyopia^71,72^, or in presbyopic subjects^70,89^. In a recent study by Lev and Polat^40^, there was large variability in the PF’s size between subjects. In addition, we noted that the masking literature reports an inter-observer variability of the experimental results^32,90,91^. Zenger and Sagi^49^ suggested that this could be accounted for if there are practice effects by the plasticity of the mechanism involved in the masking progress, indicating long-term modification in the early stage of visual processing. We suggest that heterophoria could also explain to some extent the interobserver variability found in visual psychophysical experiments in the literature.

### Monocular and binocular PFs

When compared with control subjects, the binocular horizontal PF’s size of subjects with high heterophoria showed an increase in size of 26.5%. We considered that (a) the binocular PF was also larger for the horizontal than for a vertical meridian within the phoric group, (b) heterophoria is a sensorimotor binocular disorder, and (c) monocular long-range lateral interactions of subjects presenting high heterophoria are abnormally patterned only for the horizontal meridian^17^. Thus, we wanted to assess the monocular PF’s size for the horizontal meridian.

**Figure 4:**
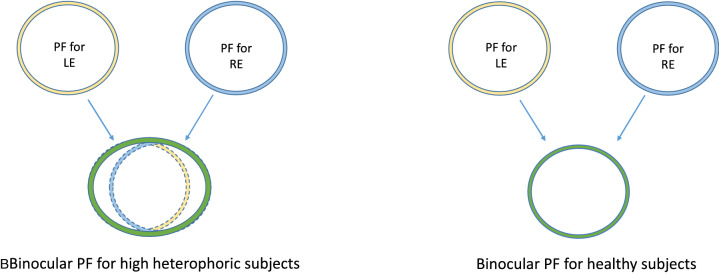
Illustration of a model explaining the larger binocular PF’s size for the horizontal meridian for subjects presenting a high heterophoria in comparison to healthy subjects.

The binocular horizontal PF was 38.6% larger than the monocular within the phoric group, whereas it was only 17.7% within the control group. Namely, this difference found in the phoric group was more than twice as large as the difference (not significant) between the binocular and the monocular PF sizes found in the control group. Nevertheless, although facilitation was significantly decreased at 3λ in monocular viewing for the phoric group^17^, the monocular PF’s size, when comparing the heterophoric and healthy subjects, was statistically similar, about the typical size found in the literature, i.e., between 2 and 3λ^30,42,49,50^. We assumed that the binocular PF’s size found for the horizontal meridian in subjects presenting a high heterophoria can be explained by the fact that they cannot maintain exactly their monocular PFs overlaid (see Fig. 4) because of a binocular instability due to their latent horizontal deviation and poor fusional reserves.

Heterophoria is a latent ocular misalignment contained by the power of fusion^10,92,93^. Binocular fusion of the images from both eyes is enabled by similar sensory inputs from both eyes (the images should be similar in clarity, size, or brightness) and by a motor component: the fusional vergence system responsible for the simultaneous movement of both eyes in opposite directions to obtain or maintain single binocular vision. A heterophoria is compensated for when the vergence system can adequately overcome its latent deviation. Low and insufficient fusional reserves could lead to binocular instability, a step before the latent misalignment of the phoria decompensates into a manifest deviation of the strabimus^93^. Binocular instability is often associated with low fusional reserves^94^ and even subjects without heterophoria can suffer from its consequences^93^, such as in dyslexia^95^. This explanation could be in accordance with previous findings of eye-tracking recordings that did not show a statistical difference in the fixation of both eyes between heterophoric and control groups nor a decompensation of the heterophoria to a strabismus during a LM experiment^17^. Among the ten subjects enrolled in this study, all presented a disorder of the fusional vergence amplitudes, usually a convergence insufficiency for the eight subjects presenting exophoria (a divergent latent misalignment) and a divergence insufficiency for the two subjects presenting esophoria (a convergent latent misalignment). Further investigation of the fixation disparity could also confirm this explanation.

### Effect of the two different mask-phase relationships on the assessment of the PF

We chose to assess the PF via the crossover point when the suppression turns to facilitation in the lateral masking experiment^32,40^. To this end, we chose to compare the results obtained with equal-phase flankers and opposite-phase flankers. The latter should provide a slightly different size of the PF: as soon as opposite-phase flankers enter the PF of the target, their contrasts are summed, canceling each other, and leading to a decrease in contrast summation (suppression) from within the PF. However, when equal-phase flankers enter the edge of the PF of the target, first, there is an increase in contrast summation (facilitation) and second a decrease in the contrast summation. With the crossover point, when suppression turns to facilitation, as estimated by the PF’s size, this may provide a slightly larger PF size.

Generally, the PF sizes obtained by LM with opposite phases were indeed larger for all groups and all meridians. Nevertheless, we obtained a similar PF pattern with the two types of LM experiments and for both GP frequencies (4 and 8 cpd): a larger horizontal PF and an asymmetric PF for the phoric group.

## Conclusion

Heterophoria affects the PF’s size similarly to meridional amblyopia but without being attributed to refraction. We suggest that these results for the phoric population may explain the large inter-individual differences found in Lev and Polat’s^51^ study and in other lateral masking literature^90,91^.

We found an asymmetry in the PF’s size. In a previous study, Levi^65^ reported that crowding is asymmetric. Since both crowding and masking are dependent on the PF’s size and since crowding is similar to masking in certain spatial and temporal conditions^40^, we suggest that heterophoria could affect the crowding. Some studies^85,96,97^ suggested that crowding sets a limit on reading speed^65^. If so, considering our recent findings, this could corroborate the difficulties in binocular reading recently found in the high heterophoric population^5^. This calls for further investigation.

Perceptual learning is well known for restoring some quality of vision in adults with amblyopia^71,72^ or presbyopia^70,89^. Since it has been suggested that perceptual learning reduces the size of the human perceptive field^88^, we would like to investigate the effect of perceptual learning on heterophoria. We believe this could potentially offer a complementary or an alternative therapy for heterophoria compared to the conventional orthoptic or vision therapy for relieving its symptoms and its consequences on daily activities.

## Data Availability

The datasets used and/or analysed during the current study available from the corresponding author on reasonable request.
